# Development and evaluation of an IgY based silica matrix immunoassay platform for rapid onsite SEB detection†

**DOI:** 10.1039/c8ra03574a

**Published:** 2018-07-16

**Authors:** J. Achuth, R. M. Renuka, K. Jalarama Reddy, M. S. Shivakiran, M. Venkataramana, K. Kadirvelu

**Affiliations:** DRDO-BU-CLS, Bharathiar University Campus Coimbatore Tamilnadu-641046 India ramana.micro@gmail.com Kadirvelukrishna@yahoo.com +0422 2428162; Freeze Drying and Animal Product Technology Division, Defence Food Research Laboratory Siddarthanagar Mysore Karnataka- 570011 India; Department of Biotechnology, Vignan's University Guntur Andhra Pradesh-522213 India

## Abstract

The present study involves immunoassay platform development based on a surface functionalized silica matrix for rapid onsite detection of Staphylococcal enterotoxin B (SEB). Silica matrix functionalization as well as the immunoassay parameters was experimentally designed and optimized through response surface methodology (RSM). Silica surface functionalization was carried out with hydrofluoric acid (HF), ammonia, 3-aminopropyl triethoxysilane (APTES) and glutaraldehyde (GA). The RSM optimized matrix functionalization parameters for HF, ammonia, APTES and GA were determined to be 10%, 40%, 20% and 10% (V/V), respectively. Antibodies for the study were generated against recombinant SEB toxin in rabbit (anti-SEB IgG) and chicken (anti-SEB IgY). Subsequently, antibodies were immobilized on the functionalized silica matrix and were further characterized by SEM and contact angle measurements to elucidate the surface uniformity and degree of hydrophilicity. The immunoassay platform was developed with anti-SEB IgG (capturing agent) and anti-SEB IgY (revealing partner). The limit of detection (LOD) of the developed platform was determined to be 0.005 μg mL^−1^ and no cross-reactivity with similar toxins was observed. Upon co-evaluation with a standard ELISA kit (Chondrex, Inc) against various field isolates, the platform was found to be on par and reliable. In conclusion, the developed method may find better utility in onsite detection of SEB from resource-poor settings.

## Abstract 2

The present study involves immunoassay platform development based on a surface functionalized silica matrix for rapid onsite detection of Staphylococcal enterotoxin B (SEB).

## Graphical abstract

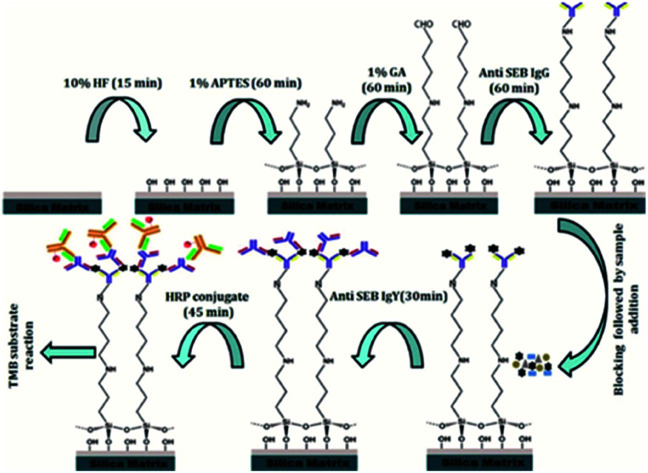

## 1. Introduction

The study of biomolecular interactions in a multi protein screening of biological/medical samples through miniaturized array-based technology is a rapidly advancing field.^1^ The silica matrix can be tailored by various surface functionalizations as well as adhesion chemistries to accommodate biomolecules *via* adsorption and covalent immobilization. The chemistry involved in the background plays a decisive role in the stability and durability of the functionalized surface.^2^ Some of the existing platforms are based on the principles of bioaffinity recognition, physisorption and covalent immobilization of biomolecules on the base substrate.^3,4^ The non-covalent attachment of molecules increases slide noise and spot background resulting in false positive results. Hence, the stable linkage involving covalent bonding between the molecules is ideal for development of robust and durable detection systems.^5,6^ The optimization of conditions for surface functionalization often significantly influences the binding properties of molecules involved and additionally could also enhance the preservation of bioprobes' native conformation/orientation.^1,7^ Functionalization of the matrix surface with amine, sulfhydryl, carboxyl and amino N hydroxyl succinamide (NHS) ester or epoxide end groups is commonly used for covalent immobilization of proteins.^3^ Glass has the capability to adsorb considerably more water than its precursor material (silicon dioxide), thus ensuring a greater surface hydration that ultimately results in accelerated silanol group formation on the surface.^8,9^ Self-assembled monolayers condense at high temperature (curing) and stabilize into siloxane linkages over the surface by cross-linking with adjacent silanols due to the absence of local water molecules.^3^ The functionalized surface becomes hydrophobic due to the presence of non-reactive alkyl groups in silane.^10^ The free amino groups project outwards producing amine functionality while protonated acidic groups orient themselves towards the glass surface.^11^

The surface activation of matrix necessitates the application of several functionalization agents that could successfully incorporate the desired functional groups. The determinations of optimum concentration for these agents under laboratory conditions are tedious and time consuming. Therefore, an *in silico* approach towards matrix functionalization overcomes such hurdles. In statistics, response surface methodology (RSM) explores the relationships between several explanatory variables and one or more response variables.^13^ RSM is a collection of mathematical and statistical techniques and will be useful for the modeling and analysis of problems in which a response of interest is influenced by several variables and the objective is to optimize this response.^12,13^ The main reason for the use of RSM encompasses the use of experimental design, generation of mathematical equations and graphics outcomes by employing multi-various factors, statistical experimental design fits into mathematical equations for prediction and optimization of factorial responses under study environment.^14^ The RSM analysis also reduces the cost and time of overall analysis by reaching the optimal values of variables with the smallest number of experiments in the shortest time duration.^15,35^ The first step involves identification of factors that affect experimental data followed by design of the experiment in order to minimize the effects of uncontrollable parameters and finally statistical analysis to separate the effects of the various factors.^16^ The criteria for the optimal design of experiments are mostly associated with the mathematical model of the process which is generally polynomials with unknown structure. The designs could be of full factorial design approach, central composite rotatable design and D optimal design, wherein the central composite rotatable design is selected in the present study.^17^ The objective of RSM in present study was to optimize the silica matrix functionalization factors and bioprobes in order to develop a sensitive, high responsive detection platform. Designed RSM models were further validated through laboratory protocols thus to develop cost-effective *in vitro* diagnostic platform for detection of SEB.

*Staphylococcus aureus* comes under the list of pathogenic organisms that poses a serious challenge during clinical infections. *S. aureus* produces a wide variety of exotoxins and related virulence factors such as cytolysins *etc.*, that alter immune function during the local infection environment.^18^ The Staphylococcal SAgs secreted in late stationary phase results in serious human illnesses, such as TSS through their effects on T lymphocyte and APC cytokine production, also SFP (Staphylococcal food poisoning) is one of the most prevalent causes of gastroenteritis worldwide caused by SE's (Staphylococcal enterotoxins).^19^ The organism is also profoundly gaining resistance to antibiotics and with the likes of α-hemolysin, several enterotoxins (from SEA to SHV), TSST and other secretory proteins it poses a serious emerging threat.^20–22^ Furthermore, SEB is one of the most potent potential agents of bioterrorism, despite its ban after Biological and Toxin Weapons Convention (BWC) of 1972, it remains as serious concern that SEB could be used as bio-warfare agent.^23^ The SEB toxin could also be aerosolized and its superantigenic nature leads to incapacitation of enemy forces.^24^ It is therefore critical to develop countermeasures to prevent or treat the lethal and incapacitating effects of SEB.^25^

In the present study, an IgY based silica matrix immunoassay platform for rapid and onsite SEB detection was developed. SEB is a potent biothreat agent with LD_50_ and ED_50_ values of 20 ng kg^−1^ and 0.4 ng kg^−1^ body weights respectively.^26^ There are numerous detection platforms available that follows enzyme-linked immunosorbent assay and bio-nano transduction principle and sensitive enough to detect 2.9 ng mL^−1^ and 10 ng mL^−1^, respectively.^27,28^ However, for onsite detection of toxin and pathogens, detection systems developed by avoiding tedious laboratory settings and skilled manpower is requisite. Moreover, a cost effective detection platform will also ensure its deployment under resource-poor settings.^29^ Unfortunately, existing detection assays involve sample pre-incubation to reduce binding of SpA to IgG that makes it tedious and time-consuming.^30^ Avian IgY antibodies doesn't have an affinity towards Staphylococcal protein A making it suitable for immunoassays that involve *S. aureus* related toxins and antigens.^31^ The avian IgY and mammalian IgG are functionally equivalent but the former has added advantage of being non-invasive, economic, convenient, and also quantitatively higher than the latter.^29^ Hence, the study was intended to develop a cost-effective, sensitive onsite SEB detection platform from food and environmental sources. In brief, a theoretical design for surface functionalization was established through RSM and its characterization was carried out by scanning electron microscopic analysis and contact angle measurements. This was further employed in development of SEB detection platform. The platform was evaluated for its sensitivity, specificity and reliability for onsite detection by assessing several pure cultures as well as naturally contaminated food samples.

## 2. Experimental

### 2.1 Materials

Microscopic glass slides and potassium dichromate were obtained from HiMedia (Mumbai, India). Hydrofluoric acid (HF), sulphuric acid, ammonia and glutaraldehyde (GA) were obtained from Merck Millipore (Bengaluru, India). 3′-Aminopropyl triethoxysilane (APTES) was obtained from Sigma Aldrich (USA). Tetra methylene benzidine–hydrogen peroxide (TMB/H_2_O_2_) was obtained from Aristogene Pvt Ltd (Bengaluru, India). Freund's complete and incomplete adjuvant, horse anti-rabbit IgG HRP and donkey anti-chicken IgY HRP were procured from Sigma Aldrich (US). Other chemicals used in the study were fine grade and obtained from Merck Millipore (Bengaluru, India).

### 2.2 Preparation and evaluation of bioprobes for immunoassay

#### 2.2.1 Generation and characterization of anti-SEB IgG

New Zealand rabbits weighing approximately of 1 kg were immunized subcutaneously with 150 μg rSEB^32^ in Freund's complete adjuvant followed by booster doses with same concentration in Freund's incomplete adjuvant at 15 days interval. Rabbits were bled from ear vein pre and post immunization (35^th^ day) and sera were collected by incubating at 37 °C for 60 min followed by centrifugation at 12 000 rpm for 5 min and stored at −20 °C until further use. The anti-SEB IgG titer was determined onto rSEB (10 μg per well) coated immunoassay plates followed by standard indirect ELISA protocols. (Ethical statement: ANUCPS/IAEC/AH/Protocol/2/2014: Dt 15/07/2014).

#### 2.2.2 Generation and characterization of anti-SEB IgY

White leghorn chickens of 22 weeks old were purchased from certified Suguna poultry (Coimbatore, India) and checked for the presence of anti-SEB IgY in the serum by indirect-ELISA method. The chickens were then immunized intramuscularly (i.m) with 150 μg of rSEB emulsified with Freund's complete adjuvant under the breast muscles. Subsequent booster immunizations were administered with an equivalent dosage of the protein emulsified with Incomplete Freund's adjuvant at an interval of 15 days.^32^ After five successive immunizations and attaining the desired immune-reactivity (1 : 8000) in sera, IgY was purified from eggs by PEG precipitation method.^33^ The anti-SEB IgY titer was determined onto rSEB (10 μg per well) coated immunoassay plates followed by standard indirect ELISA protocols.^32^

### 2.3 Preparation and characterization of silica matrix platform for immunoassay

**Fig. 1:**
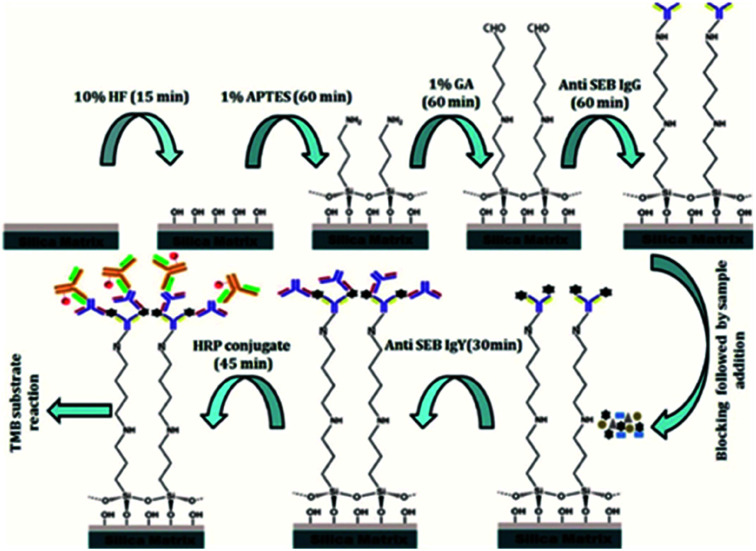
Schematic representation of the study.

Detailed flowchart for the assay development was depicted in Fig. 1.

#### 2.3.1 Design of experiment by CCD and method optimization

The parameters for silica matrix functionalization and bioprobes immobilization were optimized with central composite rotatable design (CCD) of response surface methodology with different combinations of HF, ammonia, APTES and GA levels using software State–Ease (Design Expert version 6.0.10). The experimental combinations for matrix functionalization were designed based on four independent process variables that include HF (1–40%), ammonia (1–25%), APTES (1–100%), and glutaraldehyde (1–25%) and optical density (OD) values as their responses. The bioprobes incubation onto the functionalized matrix was optimized for both capturing and revealing probes (0–60 min) with OD as their response factor. The number of design points was obtained based on the number of independent variables and these consisted 30 and 13 sets of experiments for silica matrix functionalization (Table 1) and bioprobes incubation period (Table 2), respectively.

**Table 1:** Designs of experiments for the optimization of silica matrix functionalization conditions

Run^a^	Factors				Response
	HF^b^, %	Ammonia^c^, %	APTES^d^, %	GA^e^, %	Optical value^f^
1	20.5	13	50.5	13	0.812
2	20.5	13	1	13	0.89
3	10.75	19	75.25	19	0.26
4	20.5	13	50.5	13	0.58
5	1	13	50.5	13	0.2
6	20.5	13	50.5	13	0.8
7	30.25	7	75.25	7	0.15
8	30.25	19	25.75	7	0.52
9	30.25	7	75.25	19	0.155
10	20.5	13	50.5	25	0.325
11	40	13	50.5	13	0.128
12	10.75	19	75.25	7	0.35
13	10.75	7	75.25	7	0.18
14	30.25	7	25.75	19	0.248
15	30.25	19	75.25	19	0.187
16	20.5	25	50.5	13	0.684
17	20.5	13	50.5	13	0.725
18	10.75	19	25.75	7	1.301
19	20.5	1	50.5	13	0.35
20	30.25	19	25.75	19	0.253
21	10.75	7	25.75	19	0.487
22	10.75	7	25.75	7	1.05
23	30.25	7	25.75	7	0.39
24	10.75	19	25.75	19	0.622
25	30.25	19	75.25	7	0.159
26	20.5	13	50.5	13	0.759
27	20.5	13	50.5	13	0.85
28	10.75	7	75.25	19	0.12
29	20.5	13	100	13	0.102
30	20.5	13	50.5	1	0.82

a Run Order.

b HF (1–40%).

c Ammonia (1–25%).

d APTES (1–100%).

e GA (1–25%).

f Optical density (nm), HF-hydrofluoric acid, AN-ammonia, APS-3 aminopropyl triethoxy silane, GA-glutaraldehyde.

**Table 2:** Designs of experiments for optimization of bioprobes for immunoassay

Runs^a^	Factors		Responses
	Capturing antibody^b^	Revealing antibody^b^	Optical value^c^
1	30.00	22.50	0.35
2	30.00	22.50	0.37
3	51.21	38.41	1.32
4	51.21	6.59	0.27
5	30.00	22.50	0.38
6	30.00	0.00	0.01
7	30.00	45.00	0.66
8	0.00	22.50	0.01
9	8.79	38.41	0.18
10	60.00	22.50	1.11
11	8.79	6.59	0.15
12	30.00	22.50	0.39
13	30.00	22.50	0.40

a Run Order.

b Capturing and revealing antibody (0–60 min).

c Optical density (nm).

The response from the results for the central composite rotatable designs was used to fit second-order polynomial equation. The regression analysis of the response *i.e.* reduction percentage was carried out by fitting with suitable models represented by (1) and (2). All variables of the polynomial regression at a significance level of *p* < 0.05 were included in the model, and the coefficient of determination (*r*^2^) was generated in order to assess the accuracy of the model. The response surfaces were generated from the equation of the second order polynomial, using the values of each independent variable to the maximum quadratic response.^34,45^

First order linear eqn (1)1
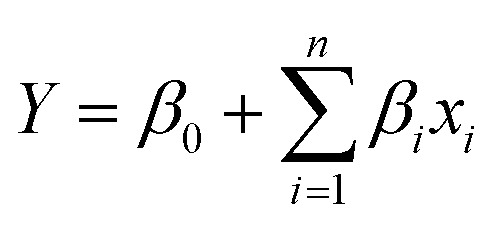


Second-order polynomial eqn (2)2
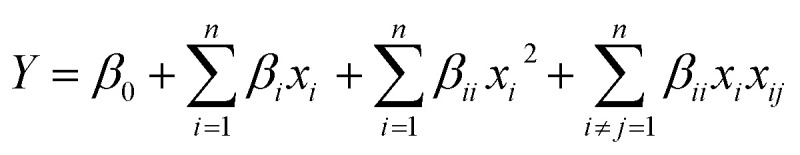
where 0 was the value of the fitted response at the centre point of the design, *i*, *ii* and *ij* were the linear, quadratic and cross product (interaction effect) regression terms respectively and *n* denoted the number of independent variables. All the designed experimental models were validated under laboratory conditions simultaneously. The incubation period for successive matrix functionalization steps were HF (30 min), ammonia wash (20–30 s), APTES (1 h)^7^ and GA (30 min)^2^ and carried out at room temperature.^36^

### 2.4 Characterization of immunoassay matrix

#### 2.4.1 Scanning electron microscopy

The immunoassay matrix developed on silica substrate was analyzed to observe the change in surface morphology at various points of chemical modifications. Scanning electron microscopic images of plain glass, HF treated (10%), HF etched glass treated with APTES (20%), silanized glass treated with glutaraldehyde (10%) and bio-functionalized with anti-SEB IgG antibody (1 : 1000) glass surfaces were obtained using FEI Quanta 200 system at 25 kV in low vacuum mode with magnification of 500× and 1000×. The substrates before analysis were sprayed with gold particles and various portions of the glass slides were analyzed to observe the uniformity in surface modification.

#### 2.4.2 Contact angle measurement

The contact angle of the glass surface was analyzed to observe the change in surface energy after subsequent modification steps. Functional groups produced on the glass surface after each modification step will be reflected based on the prevailing surface energy. Advancing and receding contact angles were measured using Kruss DSA-10E system by adding or subtracting volume to a drop and imaging when the three-phase contact line just starts to move. Images were analyzed with an in-house MATLAB based image processing code. The volume of water used was 5 μL and the contact angle was measured 5 s after the drop was deposited. Plain glass, HF treated (10%), HF etched glass treated with APTES (20%), silanized glass treated with glutaraldehyde (10%) and bio-functionalized with anti-SEB IgG antibody (1 : 1000) glass surfaces were analyzed and average of the results obtained for five different locations on the substrate is reported as final contact angle value.

### 2.5 Development of surface functionalized silica based immunoassay for detection of SEB

Following successful characterization of the functionalized silica matrix, a simple to use, cost-effective, sandwich ELISA method was developed for onsite SEB detection from food and environmental samples.

#### 2.5.1 Specificity and sensitivity evaluation of immunoassay matrix

The sensitivity of individual bioprobes (anti-SEB IgG and anti-SEB IgY) was analyzed by indirect ELISA by coating different rSEB concentrations (10 μg mL^−1^ to 0.001 μg mL^−1^) on microtiter plates. The specificity of the sandwich ELISA (SEB IgG as capturing probe and anti-SEB IgY as its revealing partner) was carried out on different toxins of *S. aureus* coated onto microtiter plates.

The specificity and sensitivity of the functionalized immunoassay matrix was carried out with anti-SEB IgG as the capturing antibody and anti-SEB IgY as its revealing partner. The specificity was evaluated on different toxins produced by *S. aureus* strains as well as the cross-reacting cell wall surface protein A and the sensitivity was evaluated onto different rSEB toxin concentration (10 μg mL^−1^ to 0.001 μg mL^−1^).

#### 2.5.2 Evaluation of immunoassay matrix on natural samples

To check the feasibility of the developed platform, different food matrixes and standard cultures were subjected to the immunoassay. Further, the intra and inter assay coefficient of variance was estimated. The solid food samples (meat and cake) were homogenized (1 g of the sample in 9 mL PBS) and centrifuged at 5000 rpm for 10 min. Similarly, the liquid food samples and overnight broth cultures were centrifuged at 5000 rpm for 10 min. The supernatant (100 μL) was analyzed with developed platform and simultaneously was co-evaluated with standard ELISA kit (Chondrex, Inc; 6214).

## 3. Results

### 3.1 Preparation and evaluation of antibodies for immunoassay

#### 3.1.1 Generation and characterization of anti-SEB IgG

The antibody titer value determined by indirect ELISA for post-immunized sera (40^th^ day) was found to be 1 : 1 28 000 and furthermore no significant reactivity were observed for the pre-immunized sera (ESI Fig. 1A†).

#### 3.1.2 Generation and characterization of anti-SEB IgY

The anti-SEB IgY extracted from hyperimmune chicken's egg yolk was found to have titer value of 1 : 32 000, whereas no reactivity was observed for the IgY extracted from pre-immunized sera as determined through indirect ELISA (ESI Fig. 1B†).

### 3.2 Preparation and characterization of silica matrix platform for immunoassay

#### 3.2.1 CCD optimization of matrix functionalization and immunoassay platform

**Fig. 2:**
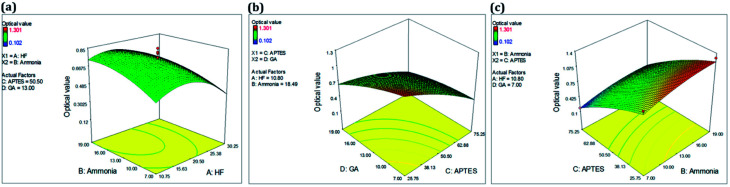
RSM analysis of silica matrix functionalization. 3D plot for optimized conditions of (a) ammonia & HF, (b) GA & APTES and (c) APTES and ammonia.

The CCRD results of RSM were used to fit the second order polynomial equation. Conversely, the regression analysis of the response (optical value) was conducted by fitting the suitable model. The effect of variations in the levels of dependent variables (HF, ammonia, APTES, GA) in the present design on three responses has been depicted as 3D response plots, cubical prediction and point prediction in Fig. 2. As illustrated in Fig. 2a, an increase in HF concentration (up to 20.50%) produced higher optical response, whereas higher concentrations led to a uniform reduction in the response factor. HF concentrations resulted in increased fragility of the final assay platform probably due to over leaching of base material (glass). Silica matrices etched with HF would activate larger number of surface hydroxyl groups that could in turn accommodate more number of silane molecules.^37^ The ammonia treatment of the matrix surface produced a steady increase in the response factor up till the maximum concentration. The other variables, APTES and GA were maintained constant at its mid values of 50.50% and 13.00%, respectively for the above experiment. As depicted in Fig. 2b, it can be deduced that, lower concentrations of both APTES and GA produced higher optical response. The other variables, HF and ammonia were maintained constant at 10.80% and 18.49% respectively, for the preceding experiment. The 3D response counter plots for ammonia and APTES (Fig. 2c) reveals that higher ammonia concentration and lower APTES concentration resulted in a better response. Herein, it can be observed that a lower concentration of HF and APTES produces higher optical response whereas higher concentration of ammonia produced better response.^38,39^ The higher concentrations of silanes are susceptible to the formation of multilayer thus prone to wash off, whereas lower concentrations such as <10% will produce thin monolayers.^3^

Silanization

(Silica matrix) OH^−^ + H_2_N(CH_2_)_3_Si(OC_2_H_5_)_3_ → OHSi(O)_3_(CH_2_)_3_NH_2_

The silanized matrix was immediately subjected to curing (heat treatment) to facilitate condensation reaction of adjacent silanol groups that would result in stable siloxane linkages which would reduce its susceptibility towards hydrolysis.^37^ Curing was carried out at a temperature of 100 °C for 5 min and precede instantly for further steps.

Cross-linking

OHSi(O)_3_(CH_2_)_3_NH_2_ + CH_2_(CH_2_CHO)_2_ → OHSi(O)_3_(CH_2_)_3_N–CH–(CH_2_)_3_CHO

**Fig. 3:**
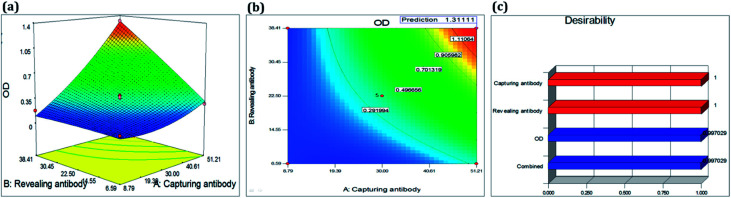
RSM analysis of bioprobe optimization. (a) 3D plot for optimized conditions of capturing and revealing antibody, (b) predicted values of capturing and revealing antibody and (c) desirability factor optimization.

The effects of different incubation period for the independent variables rabbit IgG and chicken IgY on response value (optical density) is depicted in Fig. 3a–c. The 3D response plot (Fig. 3a) reveals that a higher incubation period produced the better response. The counterplot (Fig. 3b) illustrates the different predicted optical values for the increasing incubation period (capturing and revealing probe, subsequently) with a maxima at 1.381 OD. The Fig. 3c shows the desirability factor for these analyzed bioprobes.

Biomolecule immobilization

OHSi(O)_3_(CH_2_)_3_N–CH–(CH_2_)_3_CHO + H_2_N–B (Biomolecule) → OHSi(O)_3_(CH_2_)_3_N–CH–(CH_2_)_3_–CH–N–B

The Analysis of Variance (ANOVA) was applied to fit a proper model between independent variables, response factor and to evaluate the model statistics for the optimization (Tables 3 and 4). In the present design, a quadratic model was well appropriate and selected for the optimization of independent variables (<0.0001). The predicted *R* squared value of the study was 0.75 and 0.93 for silica matrix functionalization and bioprobes standardization of immunoassays respectively. The *F* values were 17.63 and 145.59, respectively for both silica matrix functionalization and bioprobes standardization of immunoassays. The present study with large *F* value and small *p* value indicated a more significant conclusion on the corresponding response variable. The optimized designs were found to have 94% and 99% of desirability and show that both were well fitted.

**Table 3:** ANOVA & model statistics for the optimization

Term model	Responses
	Optical value
*F* value	17.63
*P* > *F*	<0.0001
Mean	0.48
S.D^a^	0.11
CV%	22.33
*R* squared	0.94
Adjusted *R* squared	0.88
Predicted *R* squared	0.75
Adequate precision	16.98
**Model**	**Quadratic**

a Standard deviation.

**Table 4:** ANOVA & model statistics for the bioprobe immobilization

Term model	Responses
	Optical density (nm)
*F* value	145.59
*P* > *F*	<0.0001
Mean	0.43
S.D^a^	0.05
CV%	11.69
*R* squared	0.99
Adjusted *R* squared	0.98
Predicted *R* squared	0.93
Adequate precision	38.78
**Model**	**Quadratic**

a Standard deviation.

Multiple regression equations generated for responses are represented as follows,

Final equation in terms of actual factors (silica matrix functionalization):Optical value = +0.90057 + 0.025415 × HF + 0.078404 × ammonia − 0.013572 × APTES − 0.031323 × GA − 5.55556 × 10^−4^ × HF × ammonia + 4.63610 × 10^−4^ × HF × APTES + 1.08547 × 10^−3^ × HF × GA − 7.15488 × 10^−5^ × Ammonia × APTES − 4.30556 × 10^−4^ × Ammonia × GA + 6.45623 × 10^−4^ × APTES × GA − 1.61451 × 10^−3^ × HF^2^ − 1.81192 × 10^−3^ × Ammonia^2^ − 1.15056 × 10^−4^ × APTES^2^ − 42650 × 10^−3^ × GA^2^

Final equation in terms of actual factors (bioprobe incubation period standardization)OD = +0.20234 − 0.013515 × capturing antibody − 4.53964 × revealing antibody + 7.46667 × capturing antibody × revealing antibody + 2.22639 × capturing antibody^2^ − 4.96296 × revealing antibody^2^

The contour plots in Fig. 2 were suitable to represent optimization process since they allow defining the optimal conditions for achieving the maximum percentage of optical response. From response surfaces, it can be observed that lower APTES and GA concentration resulted in better responses. However, a higher concentration of ammonia and medium HF levels resulted in a better response, as summarized in Table 5.

**Table 5:** Optimized values of silica based immunoassay platform

Parameters	RSM					
	Matrix optimization				Bioprobe optimization	
	HF	AN	APTES	GA	CA	RV
Concentration (a and b)	10.80^a^	18.50^a^	25.75^a^	7.00^a^	—	—
Time (in min)	—	—	—	—	51.21	38.27

a a-concentration in percentage, b-concentration in dilutions, HF-hydrofluoric acid, AN-ammonia, APS-3 aminopropyl triethoxy silane, GA-glutaraldehyde. CA-capturing antibody (anti-SEB IgG), RA-revealing antibody (anti SEB IgY).

### 3.3 Characterization of immunoassay matrix

#### 3.3.1 Scanning electron microscopy

**Fig. 4:**
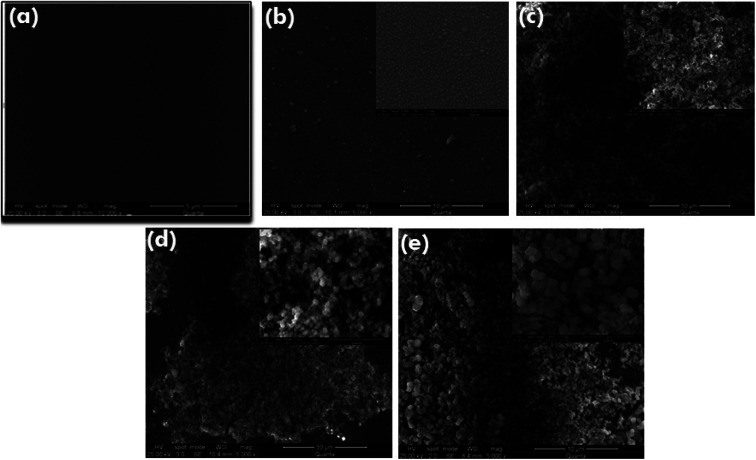
SEM characterization of functionalized matrix. The silica matrix were characterized through scanning electron microscopy for various functionalization steps (a) plain glass surface, (b) glass surface treated alone with hydrofluoric acid (10%), (c) hydrofluroic acid etched glass surface treated with 3 aminopropyl triethoxysilane (20%), (d) silanized glass surface treated with glutaraldehyde (10%) and finally (e) glass surface bio-functionalized with antibody (1 : 1000).

The SEM micrographs of the functionalized silica matrix revealed surface morphological changes after subsequent activation. The plain silica matrix micrograph (Fig. 4a) revealed no deposition or etching pattern thus assuring about proper washing before the functionalization steps. The 10% HF treated matrix micrograph (Fig. 4b) showed surface etching and subsequent treatment with 20% APTES (Fig. 4c) revealed silane deposition as spherical particle agglomeration. Consequent glutaraldehyde treatment revealed morphological changes on the spherically deposited silane particles (Fig. 4d). A uniform deposition of silane molecules throughout the silica matrix was observed upon curing (100 °C heat treatment) post APTES treatment.^38^ Therefore, the presence of such uniform reactive groups throughout the activated surface increases the proportion of bioprobes on the surface and ultimately results in better sensitivity of the assay. This bioprobe immobilization further reveals morphological changes on the agglomerated silane particles (Fig. 4e).

#### 3.3.2 Contact angle measurement

**Fig. 5:**
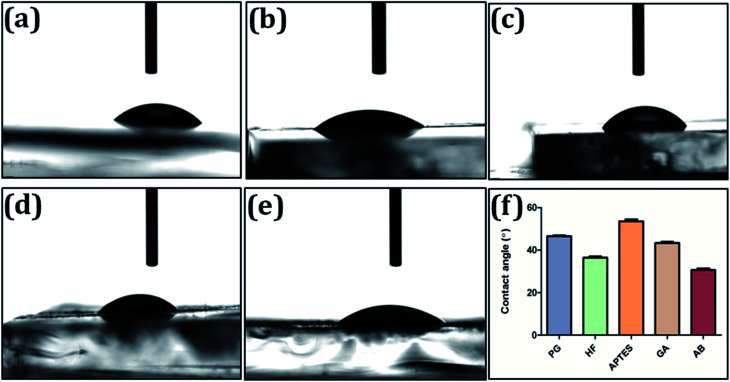
Contact angle measurement of functionalized matrix. The silica matrix were characterized by contact angle measurement for various functionalization steps (a) plain glass surface, (b) glass surface treated alone with hydrofluoric acid (10%), (c) hydrofluroic acid etched glass surface treated with 3 aminopropyl triethoxysilane (20%), (d) silanized glass surface treated with glutaraldehyde (10%) and finally (e) glass surface bio-functionalized with antibody (1 : 1000). (f) Contact angle values of plain glass (PG), hydrofluoric acid (HF), 3′ amino propyl triethoxy silane (APTES), glutaraldehyde (GA) and antibody (Ab) were analyzed with an in-house MATLAB-based image processing code. The data processed using one way-ANOVA and *p* value < 0.05 was significant.

The contact angle measurement at five different points on the surface functionalized silica matrix with uniform drop volume of 5 μL was shown in Fig. 5. Among the different activation steps, HF treatment of silica matrix resulted in contact angle of 36.43° (Fig. 5b), whereas the plain matrix had 46.56° (Fig. 5a). The decrease in contact angle could be due to the increased surface roughness upon HF treatment^9^ (Cras *et al.*, 1999). The HF treated matrix upon silanization resulted in contact angle of 53.6° (Fig. 5c) therewith indicating an increased surface hydrophobicity possibly due to the protruding free amino groups of APTES.^40,41^ Glutaraldehyde treatment preceding the silanization decreases the contact angle to 43.367° (Fig. 5d). Bioprobe immobilization of the activated silica matrix with antibody further decreases the contact angle value to 30.66° (Fig. 5e).

### 3.4 Specificity and sensitivity of developed ELISA assay

#### 3.4.1 Specificity and sensitivity of developed bioprobes

The individual bioprobes allowed the detection at 0.005 μg mL^−1^ for both rabbit anti-SEB IgG and chicken anti-SEB IgY (ESI Fig. 2A†). The individual bioprobes assessed for specificity revealed that rabbit anti-SEB IgG cross-reacted with the protein A of *Staphylococcus aureus* whereas the chicken anti-SEB IgY showed cross-reactivity towards any other toxins. The sandwich ELISA was found to detect SEB specifically and prominently than anti-SEB IgY indirect ELISA antibody-based indirect ELISA (ESI Fig. 2B†).

#### 3.4.2 Specificity and sensitivity of developed immunoassay matrix

**Fig. 6:**
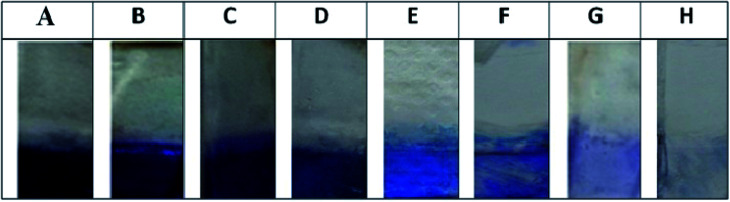
Sensitivity of the immunoassay matrix. Sensitivity of the functionalized matrix were carried out by incubating the bioconjugated matrix with decreasing concentration of SEB toxin (A) 10 μg mL^−1^, (B) 5 μg mL^−1^, (C) 1 μg mL^−1^,(D) 0.5 μg mL^−1^, (E) 0.25 μg mL^−1^, (F) 0.1 μg mL^−1^, (G) 0.05 μg mL^−1^, (H) 0.01 μg mL^−1^ and were further probed with anti SEB IgY.

**Fig. 7:**
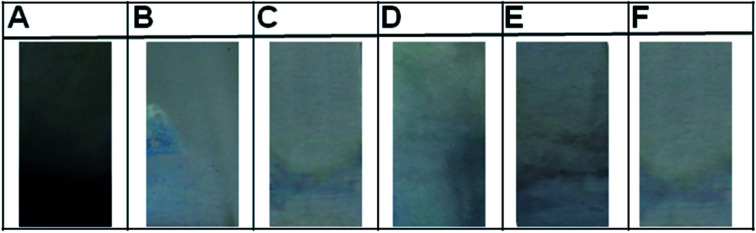
Specificity of the immunoassay matrix. Specificity of the functionalized matrix were assayed by incubating bioconjugated matrix with various *Staphylococcal aureus* toxins (A) Staphylococcal enterotoxin B, (B) Staphylococcal enterotoxin A, (C) Staphylococcal enterotoxin C, (D) toxic shock syndrome toxin 1, (E) protein A and (F) negative control (PBS).

The sensitivity of the immunoassay matrix generated was performed with various concentrations of antigen (rSEB) ranging from 10 μg mL^−1^ to 0.001 μg mL^−1^ with anti-SEB IgG (1 : 1000 for 60 min) capturing and anti-SEB IgY (1 : 100 for 30 min) as revealing probe (Fig. 6). The lowest detection value of the developed sandwich platform was determined as 0.005 μg mL^−1^ of SEB antigen which is well below the LD_50_ value. The developed method showed no cross-reactivity with other related enterotoxins and protein A (Fig. 7).

### 3.5 Evaluation of developed sandwich ELISA on food samples

The evaluation of individual bioprobes onto natural samples and standard cultures by indirect ELISA (with anti-SEB IgG and anti-SEB IgY) as well as sandwich ELISA are given in ESI Fig. 3.† The indirect ELISA by anti-SEB IgG resulted in false positive reactions in food isolates and standard cultures that are negative for SEB as shown in ESI Fig. 3A (MI 01), B (MT01 and 05), C (CK 02, 03, 08 and 09) and D (B-*S. aureus* ATCC 19095).† These results point to the fact that mammalian IgG cross-reacts with Staphylococcal protein A producing false positive results in non SEB positive *Staphylococcus aureus* cultures as well as SEB negative food isolates. The indirect ELISA with anti-SEB IgY and sandwich ELISA detects only the SEB positive samples specifically.

**Fig. 8:**
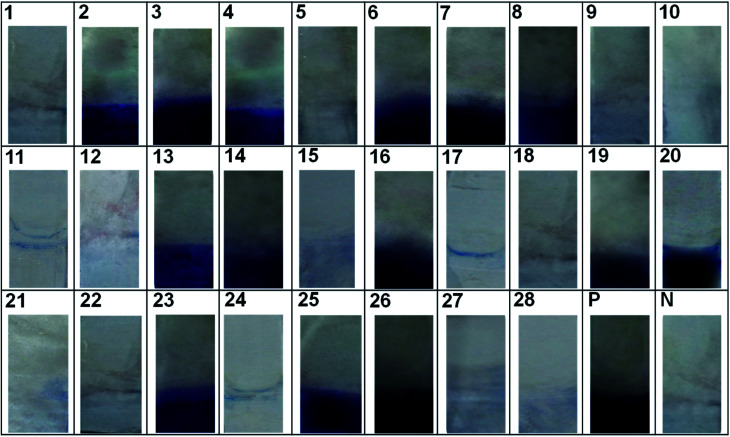
Evaluation of the immunoassay matrix. Evaluation on to field samples and standard cultures, (1) MI 01, (2) MI 02, (3) MI 03, (4) MI 04, (5) MT 01, (6) MT 02, (7) MT 03, (8) MT 04, (9) MT 05, (10) CK 01, (11) CK 02, (12) CK 03, (13) CK 04, (14) CK 05, (15) CK 06, (16) CK 07, (17) CK 08, (18) CK 09, (19) CK 10, (20) *S. aureus* ATCC-29213, (21) *S. aureus* ATCC-19095(SEC positive), (22) *S. epidermidis* ATCC-12228, (23) *S. aureus* NCIM-5021, (24) *Salmonella typhimurium* ATCC-14028, (25) *S. aureus* NCIM-2657, (26) *S. aureus* NCIM-2654, (27) *Escherichia coli* ATCC-10536, (28) *Klebsiella pneumonia* ATCC-10031, (P) positive control (rSEB protein) and (N) negative control (PBS).

To check the reliability and field usage, developed method was evaluated on to various contaminated food samples and reference cultures. The results of the developed immunoassay were on par with the standard ELISA kit method (Table 6 and Fig. 8).

**Table 6:** Co-evaluation of developed matrix platform with standard ELISA kit

S. no	Source	Chondrex standard ELISA kit	Developed IgY based silica matrix platform
1	Milk isolate 01	−	−
2	Milk isolate 02	+	+
3	Milk isolate 03	+	+
4	Milk isolate 04	+	+
5	Meat isolate 01	−	−
6	Meat isolate 02	+	+
7	Meat isolate 03	+	+
8	Meat isolate 04	+	+
9	Meat isolate 05	−	−
10	Cake isolate 01	−	−
11	Cake isolate 02	−	−
12	Cake isolate 03	−	−
13	Cake isolate 04	+	+
14	Cake isolate 05	+	+
15	Cake isolate 06	−	−
16	Cake isolate 07	+	+
17	Cake isolate 08	−	−
18	Cake isolate 09	−	−
19	Cake isolate 10	+	+
20	*S. aureus* ATCC-29213	+	+
21	*S. aureus* ATCC-19095(SEC positive)	−	−
22	*S. epidermidis* ATCC-12228	−	−
23	*S. aureus* NCIM-5021	+	+
24	*Salmonella typhimurium* ATCC-14028	−	−
25	*S. aureus* NCIM-2657	+	+
26	*S. aureus* NCIM-2654	+	+
27	*Escherichia coli* ATCC-10536	−	−
28	*Klebsiella pneumonia* ATCC-10031	−	−

Additionally, the inter as well as intra assay coefficient of variance was determined wherein it was found to between 8.9–12.6% and 3–8.4%, respectively (Table 7). Therefore, this suggests that the present method is well suited and applicable for detection of SEB from diverse sample types.

**Table 7:** The intra and inter assay coefficient of variation of the assay

S. no	Sample	Intra assay coefficient of variation (%)			Inter assay coefficient of variation (%)		
		IgG^a^	IgY^a^	Sandwich	IgG^a^	IgY^a^	Sandwich
1	Milk isolate 01	7.1	5.9	3.6	11.6	10.5	9.3
2	Milk isolate 02	7.9	6.7	4.4	12.4	11.3	10.1
3	Milk isolate 03	7.1	5.9	3.6	11.3	10.2	9
4	Milk isolate 04	7.6	6.4	4.1	11.5	10.4	9.2
5	Meat isolate 01	7.3	6.1	3.8	11.25	10.15	8.95
6	Meat isolate 02	7.8	6.6	4.3	11	9.9	8.7
7	Meat isolate 03	8.1	6.9	4.6	12.34	11.24	10.04
8	Meat isolate 04	8.4	7.2	4.9	12.6	11.5	10.3
9	Meat isolate 05	7.5	6.3	4	11.4	10.3	9.3
10	Cake isolate 01	7.8	6.6	4.3	11.8	10.7	9.5
11	Cake isolate 02	7.7	6.5	4.2	11.65	10.54	9.34
12	Cake isolate 03	7.3	6.1	3.8	11.3	10.2	9
13	Cake isolate 04	8.3	7.1	4.8	12.4	11.3	10.1
14	Cake isolate 05	6.1	4.9	2.6	11.12	10.02	8.92
15	Cake isolate 06	6.3	5.2	2.9	11.2	10.1	8.9
16	Cake isolate 07	6.8	5.6	3.3	11.7	10.6	9.4
17	Cake isolate 08	6.5	5.3	3	11.5	10.4	9.2
18	Cake isolate 09	6.8	5.6	3.3	11.6	10.5	9.3
19	Cake isolate 10	6.2	5	3	11.2	10.1	8.9

a Indirect ELISA.

## 4. Discussion

Immobilization of biomolecules onto various surfaces have been of prime focus to many researchers for development of novel, durable, ready to use and portable detection systems. To accomplish this, there are several parameters that influence surface activation strategies such as function stabilization, structure/functional group conservation and proper binding orientation of the biomolecules.^42^ The covalent attachment of biomolecules can be accomplished through variety of functionalization chemistry that imparts groups such as NH_2_, SH, COOH, NHS ester as well as epoxide and often this is achieved on a glass or oxide surface through self-assembly of silanes.^43^ The utilization of single silane ensures functional uniformity whereas a mixture of silanes could possibly result in uncharacteristic functional groups.^11^ The multilayer formation leads to an unstable silane layer, hence are vulnerable to get washed away during common washing steps of immunoassay.^3^ Therefore, the matrix functionalization in the present work is accomplished through silanization using (3-aminopropyl) triethoxysilane (APTES) on silica matrix to produce a thin and stable silane layer after hydrofluoric acid etching, upon which biomolecule immobilization was achieved *via* glutaraldehyde linker. Thus, this functionalization strategy satisfies the parameters of efficient bioprobe immobilization and non hindrance with its biological function therewith enhancing the sensitivity of assay.^44,45^ The previous studies reported till now majorly focus on glass surface activation for biomolecule immobilization^3,9,37–39,41,42^ and some pertains to assay development,^46,47^ however, none have quite evolved into a sensitive detection platform for routine laboratory application and onsite screening. Thus, the present study focuses on the development of silica functionalized matrix based onsite SEB detection platform.

Immunodetection platforms are cost effective, more suitable, robust and portable for onsite detection due to its less technical expertise requirement compared to PCR assays.^48^ The PCR assays are more sensitive as well as specific in detection of toxin associated genes but more clinical relevance could only be established through immunoassays. Moreover, the main drawback associated with PCR is their inability to correlate with the toxin expression by the organism in the samples.^32^ Therefore, the bioligands were generated with high sensitivity in rabbit and chicken systems. The sandwich immunoassay strategy was employed, wherein the anti-rabbit SEB IgG was used as the capturing probe and chicken anti-SEB IgY as its revealing partner. The anti-SEB IgG were then permanently immobilized on to the immunoassay matrix prepared through silane glutaraldehyde chemistry. Characterization by scanning electron microscopy and contact angle measurements revealed the successful matrix functionalization and bioligands immobilization onto the matrix.

The chicken egg yolk antibodies are more hygienic, cost-efficient and convenient compared with the traditional antibodies obtained from mammalian serum.^49^ The maintenance costs for keeping hens are also lower than those for mammals such as rabbits and even more viable in ethical aspect due to the non invasive purification of antibodies. Moreover, one immunized chicken could generate yield more than 22 500 mg of IgY per year that is equivalent to the production by 4.3 rabbits over a year.^50^ Furthermore, an added advantage arises because of the phylogenetic distance as well as genetic background between birds and mammals that improve the likelihood of an immune response against antigens or epitopes that may be non-immunogenic in mammals. The mammalian immunoglobulins may have deleterious effects on the performance of different immunoassay formats, particularly in their use as bioactive molecules to capture or detect the analyte, that are affected by heterophilic antibodies as well as high levels of non-specific binding (*e.g.* Staphylococcal protein A).^51^ The various approaches to eradicate heterophilic antibody interference includes removal or inactivation of interfering immunoglobulins through precipitation with PEG, buffer additives and proteolytic Fc fragments cleavage, however, these are practicably unviable for onsite detection system.^50^ This was further confirmed through indirect ELISA, wherein anti-SEB IgY antibody was not found to produce any cross reactivity unlike its mammalian counterpart anti-SEB IgG antibody without sample pre-treatment. Thus considering these factors, chicken IgY antibodies offer several advantages over mammalian counterparts as they do not interact with rheumatoid factor (RF), human anti-mouse IgG antibodies (HAMA), complement components or mammalian Fc receptors^31^ and thereby enhances the aptness of the developed platform for onsite applications.

Several SEB detection assays,^23,24,48^ as well as systems with IgY based strategies have been reported previously.^52–54^ Despite establishment of such novel approaches and numerous improvements within these described assays, the commercially available kits still utilize polyclonal/monoclonal IgG antibodies based ELISA as represented in Table 8.

**Table 8:** Commercially available SEB detection kits/platforms

Kit name	Manufacturer	Sensitivity (ng mL^−1^)	Assay time	Enrichment/extraction	Detection method
Ridascreen® SET	R-Biopharm	0.25 (liquid sample), 0.375 (solid sample), 0.25 (culture supernatant)	Within 4 h	Yes	ELISA^a^
Tecra visual immunoassay (VIA™)	3M	1	Within 4 h	Yes	ELISA
SET-RPLA toxin detection kit	Oxoid	0.5	24 h	Yes	RPLA^b^
Transia PLATE staphylococcal enterotoxins	Biocontrol	0.02	Within 2 h	Yes	ELISA
Vidas SET2	Biomerieux	0.025	Max 80 min	Yes	ELFA^c^ (automated system)
BADD SEB	AdVnt's	10	Within 15 min	No	LFA^d^
SEB antibody assay kit	Chondrex	1 (only liquid samples)	Within 2 h	Yes	ELISA
Developed silica platform	—	5	Max 90 min	No	ELISA

a ELISA-Enzyme Linked Immunosorbent Assay.

b RPLA-Reversed Passive Latex Agglutination.

c ELFA-Enzyme Linked Immunofluorescent Assay.

d LFA-Lateral Flow Assay.

The limit of detection of these represented assays range between 0.02 ng mL^−1^ to 10 ng mL^−1^. However, some of these assays are either time consuming (sample processing includes; pre-enrichment/extraction step), require sophisticated instrumentation (ELISA reader, automated system), or even labor-intensive (require technical expertise for analysis). Notwithstanding, specificity of these commercial kits is relatively low since the likelihood of false positives occurring due to matrix components (*e.g.*, protein A) with the Fc fragment (and, to a lesser extent, Fab fragments) in immunoglobulin G from several animal species (*e.g.*, mouse or rabbit, but not rat or goat) is reasonably high.^31^ Moreover, SEB being a potent bio-threat agent necessitates the detection platform to be onsite/field deployable.

Considering the above mentioned aspects, the present study has developed IgY based silica matrix platform for SEB detection. Herein, the matrix functionalization (HF, ammonia, APTES and GA) optimized through RSM technique accomplished uniform reactive groups throughout the activated surface thereby increasing the bioprobes proportion. This significantly improvised the sensitivity of the assay with a limit of detection up-to 0.005 μg mL^−1^. Likewise, application of anti-SEB IgY as revealing bioprobe enhanced specificity of the assay as no cross reactivity towards any closely associated toxins as well as other interfering factors was observed. Further, its onsite feasibility was established through SEB detection from various food matrices. Besides this, inter and intra assay coefficient of variance confirmed reproducibility of the platform. Therefore, these attributes render the developed platform to be highly specific, easy to operate, low cost, and sensitive assay for the rapid and reproducible on-site detection of SEB toxin.

## 5. Conclusion

In collective, the study presents silica matrix functionalization strategy through RSM approach, and further development of an IgY based rapid onsite SEB detection platform. The functionalization chemistry was optimized to self-assemble silane monolayers uniformly, which in turn was critical for successful homogenous biomolecule immobilization. This was further substantiated through SEM and contact angle characterization. The LOD of the developed platform was estimated to be 5 ng mL^−1^ with total assay duration of 90 min without sample processing. The robustness and on site portability of the system was verified through SEB detection from different food matrices, wherein inter and intra assay coefficient of variance was observed to be below 15% and 10%, respectively. In addition to this, the developed platform was found to be on par upon co-evaluation with commercial SEB detection kit. Therefore, the developed platform possesses high sensitivity and nil cross reactivity, thereupon confirming its significant potential for the rapid and sensitive onsite detection of SEB toxin.

## Conflicts of interest

There is no financial conflict of interest.

## Ethical statement

All animal experiments were reviewed and approved by animal ethical committee at the Acharya Nagarjuna University, Guntur, India and were conducted in accordance with the Institutional Animal Ethical Committee (IAEC) guidelines. ANUCPS/IAEC/AH/Protocol/2/2014: Dt 15/07/2014.

## Supplementary Material

RA-008-C8RA03574A-s001

## References

[cit1] Xu Q., Lam K. S. (2003). BioMed Res. Int..

[cit2] Agarwal D. K., Maheshwari N., Mukherji S., Rao V. R. (2016). RSC Adv..

[cit3] Gunda N. S. K., Singh M., Norman L., Kaur K., Mitra S. K. (2014). Appl. Surf. Sci..

[cit4] Bhakta S. A., Evans E., Benavidez T. E., Garcia C. D. (2015). Anal. Chim. Acta.

[cit5] Kamra T., Chaudhary S., Xu C., Johansson N., Montelius L., Schnadt J., Ye L. (2015). J. Colloid Interface Sci..

[cit6] Seurynck-Servoss S. L., White A. M., Baird C. L., Rodland K. D., Zangar R. C. (2007). Anal. Biochem..

[cit7] Sterzynska K., Budna J., Frydrych-Tomczak E., Hreczycho G., Malinska A., Maciejewski H., Zabel M. (2014). Folia Histochem. Cytobiol..

[cit8] Hashizume M., Fukagawa S., Mishima S., Osuga T., Iijima K. (2016). Langmuir.

[cit9] Cras J. J., Rowe-Taitt C. A., Nivens D. A., Ligler F. S. (1999). Biosens. Bioelectron..

[cit10] Kusnezow W., Jacob A., Walijew A., Diehl F., Hoheisel J. D. (2003). Proteomics.

[cit11] Seo J. H., Chen L.-J., Verkhoturov S. V., Schweikert E. A., Revzin A. (2011). Biomaterials.

[cit12] Zhang Q., Chen T., Yang S., Wang X., Guo H. (2013). Appl. Microbiol. Biotechnol..

[cit13] Montgomery S. P., Drouillard J. S., Sindt J. J., Greenquist M. A., Depenbusch B. E., Good E. J., Loe E. R., Sulpizio M. J., Kessen T. J., Ethington R. T. (2005). J. Anim. Sci..

[cit14] Barmpalexis P., Kanaze F. I., Georgarakis E. (2009). J. Pharm. Biomed. Anal..

[cit15] Tsapatsaris S., Kotzekidou P. (2004). Int. J. Food Microbiol..

[cit16] Margaritelis N. G., Markopoulou C. K., Koundourellis J. E. (2013). Anal. Methods.

[cit17] Reddy K. J., Pandey M. C., Harilal P. T., Radhakrishna K. (2013). Int. Food Res. J..

[cit18] Imani Fouladi A., Choupani A., Fallah Mehrabadi J. (2011). Kowsar Med. J..

[cit19] Cook E., Wang X., Robiou N., Fries B. C. (2007). Clin. Vaccine Immunol..

[cit20] Brosnahan A. J., Schlievert P. M. (2011). FEBS J..

[cit21] Kamboj D. V., Goel A. K., Singh L. (2006). Def. Sci. J..

[cit22] Llewelyn M., Cohen J. (2002). Lancet Infect. Dis..

[cit23] Xu Y., Huo B., Sun X., Ning B., Peng Y., Bai J., Gao Z. (2018). RSC Adv..

[cit24] Sharma A., Rao V. K., Kamboj D. V., Upadhyay S., Shaik M., Shrivastava A. R., Jain R. (2014). RSC Adv..

[cit25] Karauzum H., Chen G., Abaandou L., Mahmoudieh M., Boroun A. R., Shulenin S., Devi V. S., Stavale E., Warfield K. L., Zeitlin L., others (2012). J. Biol. Chem..

[cit26] Cook E., Wang X., Robiou N., Fries B. C. (2007). Clin. Vaccine Immunol..

[cit27] Hale M. L. , in Bioterrorism, InTech, 2012

[cit28] Venkataramana M., Kurkuri M. D., others (2016). Sens. Actuators, B.

[cit29] Sapsford K. E., Francis J., Sun S., Kostov Y., Rasooly A. (2009). Anal. Bioanal. Chem..

[cit30] Reddy P. N., Nagaraj S., Sripathy M. H., Batra H. V. (2015). Ann. Microbiol..

[cit31] Araújo A. S., Lobato Z. I. P., Chávez-Olórtegui C., Velarde D. T. (2010). Toxicon.

[cit32] Mudili V., Makam S. S., Sundararaj N., Siddaiah C., Gupta V. K., Rao P. V. L. (2015). Sci. Rep..

[cit33] Polson A., von Wechmar M. B., Van Regenmortel M. H. V. (1980). Immunol. Commun..

[cit34] Myers R. H. , Montgomery D. C., Vining G. G. and Robinson T. J., Generalized linear models: with applications in engineering and the sciences, John Wiley & Sons, 2012, vol. 791

[cit35] Sin H. N., Yusof S., Hamid N. S. A., Rahman R. A. (2006). J. Food Eng..

[cit36] Oh G.-J., Yoon J.-H., Vu V. T., Ji M.-K., Kim J.-H., Kim J.-W., Yim E.-K., Bae J.-C., Park C., Yun K.-D., others (2017). J. Nanosci. Nanotechnol..

[cit37] Gruian C., Vanea E., Simon S., Simon V. (2012). Biochim. Biophys. Acta, Proteins Proteomics.

[cit38] Metwalli E., Haines D., Becker O., Conzone S., Pantano C. G. (2006). J. Colloid Interface Sci..

[cit39] Vistas C. R., Águas A. C. P., Ferreira G. N. M. (2013). Appl. Surf. Sci..

[cit40] Chaudhary S., Kamra T., Uddin K. M. A., Snezhkova O., Jayawardena H. S. N., Yan M., Montelius L., Schnadt J., Ye L. (2014). Appl. Surf. Sci..

[cit41] Nagare G. D., Mukherji S. (2009). Appl. Surf. Sci..

[cit42] González-González M., Bartolome R., Jara-Acevedo R., Casado-Vela J., Dasilva N., Matarraz S., García J., Alcazar J. A., Sayagues J. M., Orfao A., others (2014). Anal. Biochem..

[cit43] Seo J. H., Shin D.-S., Mukundan P., Revzin A. (2012). Colloids Surf., B.

[cit44] Gang A., Gabernet G., Renner L. D., Baraban L., Cuniberti G. (2015). RSC Adv..

[cit45] Majumder O., Bankoti A. K. S., Kaur T., Thirugnanam A., Mondal A. K. (2016). RSC Adv..

[cit46] Seo J. H., Chen L.-J., Verkhoturov S. V., Schweikert E. A., Revzin A. (2011). Biomaterials.

[cit47] Antoniou A., Herlem G., André C., Guillaume Y., Gharbi T. (2011). Talanta.

[cit48] Pauly D., Kirchner S., Stoermann B., Schreiber T., Kaulfuss S., Schade R., Zbinden R., Avondet M.-A., Dorner M. B., Dorner B. G. (2009). Analyst.

[cit49] Xu Y., Li X., Jin L., Zhen Y., Lu Y., Li S., You J., Wang L. (2011). Biotechnol. Adv..

[cit50] Schade R., Calzado E. G., Sarmiento R., Chacana P. A., Porankiewicz-Asplund J., Terzolo H. R., others (2005). Altern. Lab. Anim..

[cit51] Spillner E., Braren I., Greunke K., Seismann H., Blank S., du Plessis D. (2012). Biologicals.

[cit52] Reddy P., Ramlal S., Sripathy M. H., Batra H. V. (2014). J. Immunol. Methods.

[cit53] Vinayaka A. C., Muthukumar S. P., Thakur M. S. (2013). Bionanoscience.

[cit54] Jin W., Yamada K., Ikami M., Kaji N., Tokeshi M., Atsumi Y., Mizutani M., Murai A., Okamoto A., Namikawa T., others (2013). J. Microbiol. Methods.

