# Supplementary material for: Development and evaluation of an IgY based silica matrix immunoassay platform for rapid onsite SEB detection
Source: RSC Adv. 2018 Jul 16;8(45):25500–13. doi: 10.1039/c8ra03574a (PMC9097597; doi:10.1039/c8ra03574a)
Supplement: RA-008-C8RA03574A-s001 [file RA-008-C8RA03574A-s001.pdf]

Electronic Supplementary Material (ESI) for RSC Advances.
This journal is © The Royal Society of Chemistry 2018

Supplementary Information

Title of the manuscript:

Surface functionalized silica based immunoassay for onsite detection of SEB from food and environmental samples

Authors: Achuth, J[1]., Jalarama Reddy, K[2]., Shivakiran.M.S[3]., Venkataramana. M[1]*., Kadirvelu. K[1].

Address:

1- DRDO-BU-CLS, Bharathiar University Campus, Coimbatore, Tamilnadu-641046, India.
2- Freeze Drying and Animal Product Technology Division, Defence Food Research Laboratory, Siddarthanagar, Mysore, Karnataka- 570011, India.
3- Department of Biotechnology, Vignan's University, Guntur, Andhra Pradesh-522213, India.

Address for the correspondence:

Dr. Venkataramana. M
Research Scientist
DRDO-BU-CLS
Bharathiar University Campus
Coimbatore, Tamilnadu-641046, India.
Email: ramana.micro@gmail.com
Phone: 0422 2428162

Supplementary figures:

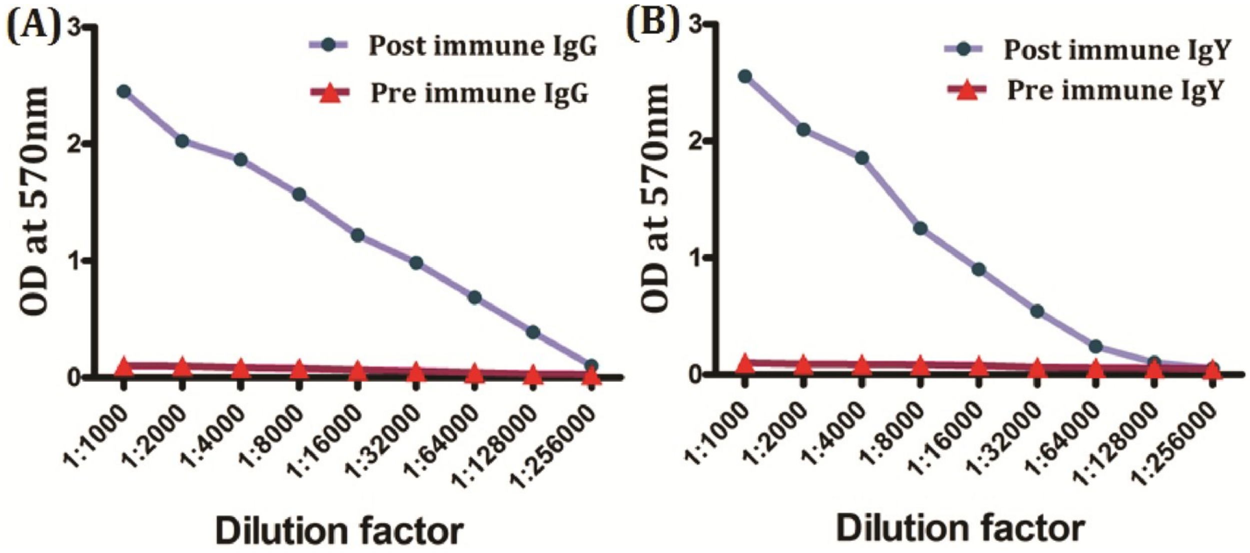

SIFig. 1: Titer value determination of anti SEB IgG and anti SEB IgY. Specific antibody level present in pre and post immune **(A)** sera and **(B)** egg yolk were analyzed through indirect ELISA against rSEB as antigen.

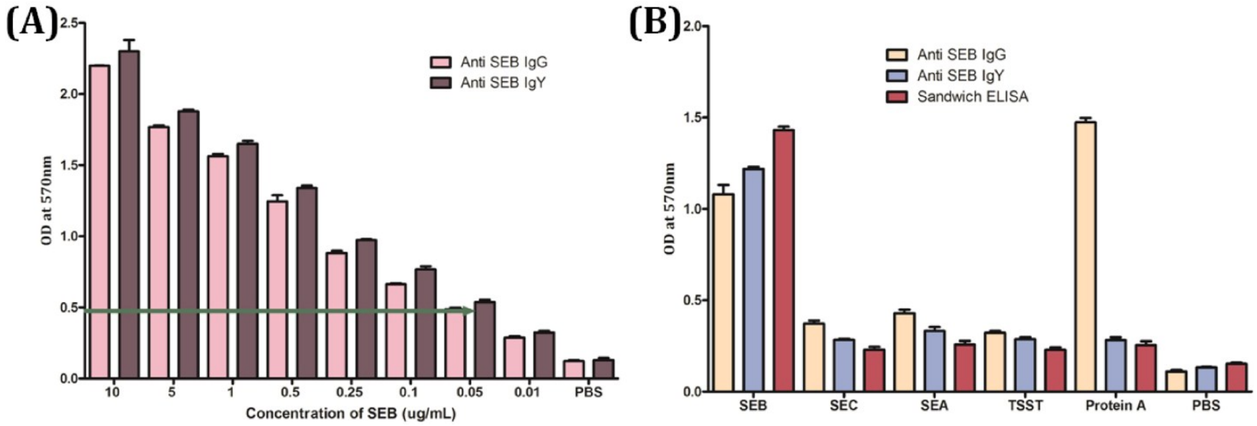

SIFig. 2: Sensitivity and specificity analysis of bioprobes. (A) The microtiter plates coated with decreasing concentration of SEB toxin (10µg/mL to 0.01 µg/mL and were further probed separately with rabbit anti SEB IgG and chicken anti SEB IgY antibodies indirect ELISA. **(B)** The microtiter plates coated with different *Staphylococcal aureus* toxins Staphylococcal enterotoxin B (SEB), Staphylococcal enterotoxin C (SEC), Staphylococcal enterotoxin A (SEA), Toxic shock syndrome toxin 1 (TSST), Protein A (SPA) and PBS (blank) and indirect ELISA performed with rabbit anti SEB IgG and chicken anti SEB IgY antibodies.

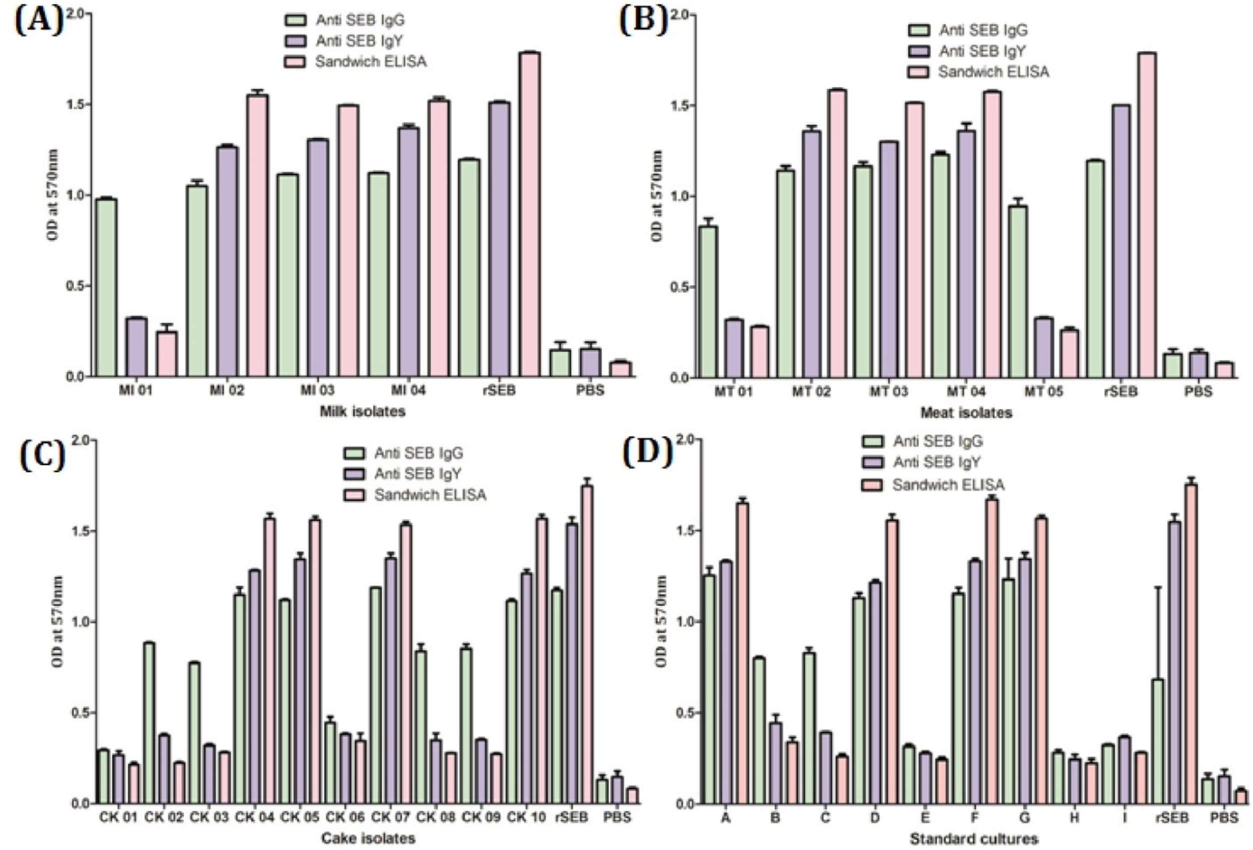

SIFig. 3: Evaluation of bioprobes. The processed food samples corresponding to **(A)** milk isolates, **(B)** meat isolates, **(C)** cake isolates and **(D)** standard cultures (A- *S. aureus* ATCC-29213, B- *S. aureus* ATCC-19095(SEC positive), C- *S. epidermidis* ATCC-12228, D- *S. aureus* NCIM-5021, E- *Salmonella typhimurium* ATCC-14028, F- *S. aureus* NCIM-2657, G- *S. aureus* NCIM-2654, H- *Escherichia coli* ATCC-10536, I- *Klebsiella pneumonia* ATCC-10031) evaluated with rabbit anti SEB IgG and chicken anti SEB IgY antibodies by indirect ELISA as well as by sandwich ELISA wherein the formed served as capturing probe and the latter as revealing probe.
